# Effect of Tahiti lime (*Citrus latifolia*) juice on the Production of the PGF2α/PGE2 and Pro-Inflammatory Cytokines involved in Menstruation

**DOI:** 10.1038/s41598-020-63477-8

**Published:** 2020-04-27

**Authors:** Thaiane Robeldo, Edione Fatima Canzi, Priscila Maria de Andrade, Jhonne Pedro Pedotte Santana, Felipe Roberti Teixeira, Valentine Spagnol, Beatriz Helena Lameiro Noronha Sales Maia, Maristela Carbol, Erika Gonçalves Caneira, Maria Fátima das Graças Fernandes Da Silva, Ricardo Carneiro Borra

**Affiliations:** 1 Department of Genetics and Evolution, Federal University of São Carlos, São Carlos, SP Brazil; 2 Department of Chemistry, Federal University of Paraná, Polytechnic Center, Curitiba, PR Brazil; 3 Department of Chemistry, Federal University of São Carlos, São Carlos, SP Brazil; 4 Department of Medicine, Federal University of São Carlos, São Carlos, SP Brazil

**Keywords:** Inflammation, Effectors in plant pathology

## Abstract

Tahiti lemon juice (*Citrus latifolia*) (TLJ), as a natural source of flavonoids, has been used as an alternative to anti-inflammatory drugs for the treatment of dysmenorrhea and menstrual excessive bleeding, often associated with an imbalance of the prostaglandins (PG) levels. However, despite the positive effects, the mechanisms that rule menstruation control are still unknown. Therefore, the objectives were to characterize the TLJ and analyze its effect on the production of PGF2α, PGE2 and pro-inflammatory cytokines involved inmenstruation. Flavonoids from TLJ were discriminated by UPLC-DAD-MS/MS (Qq-TOF) and the effects of TLJ were studied *in vitro* by quantification of the contraction of myoblasts in culture and PGF2α and PGE2 productions. Further, the systemic and menstrual fluid levels of PGF2α, PGE2, IL-1β, TNF-α, IL-6, AK1B1 and AK1C3 enzymes produced by women during the menstrual period were compared after exposition or not to TLJ or meloxicam. The results showed that TLJ induces an increase in the contraction of myoblasts and the PGF2α supernatant level. Regarding *in vivo* analysis, a higher concentration of PGF2α and an unaltered PGE2 level was also found in the menstrual blood of women treated with TLJ, in contrast with a lower level of PGE2 and PGF2α observed in the meloxicam group. Concerning cytokines, only menstrual TNF-α levels decrease after treatment with TLJ or meloxicam. In conclusion, TLJ may favor the control of menstruation events via a PGF2α mediated muscle contractile response.

## Introduction

Today’s women experience more menstrual cycles than their predecessors. In the past, late menarche, multiple pregnancies, longer periods of breastfeeding and the occurrence of menopause at 40 years of age acted as inhibiting factors of the menstruation. However, due to lifestyle changes combined with longer life expectancies and menostasis at 45 years of age, modern women are more predisposed to developing menstrual disorders characterized by prolonged and excessive bleeding along with dysmenorrhea, which compromise health and the quality of life^1,2^.

Studies have shown that both excessive menstrual bleeding and primary dysmenorrhea are related to an imbalance in the uterine concentration of the F2α (PGF2α) and E2 (PGE2) prostaglandins^3–6^. In women with dysmenorrhea, the increased PGF2α production has been associated with the sensitivity of the muscle fibers in the uterus due to impaired blood flow caused by strong contractions of the myometrium^7,8^. PGF2α is a potent vasoconstrictor that acts directly on smooth muscle fibers, reducing blood vessel caliber^9^. In the uterus, PGF2α, produced by the endometrium, acts as an agonist in the myometrium, inducing contractions by activating FPs receptors and mobilizing intracellular Ca^2+^. Meanwhile, the PGE2 activity in the myometrium depends on the type of receptor-activated. The PGE2 binding to EP1 and mobilizing the intracellular Ca^2+^, or to EP3 and modulating cAMP and IP3, produces contractions. On the other hand, the binding of PGE2 to EP2 or EP4 induces relaxation via elevating intracellular cAMP accumulation^10–12^. The direct involvement of prostaglandins in the pain pathways associated with menstrual disorders, mainly the PGE2, should notbe discarded^8^.

Depending on the magnitude of the menstrual disorder, non-steroidal anti-inflammatory drugs are used to block the synthesis of PGs and control the clinical conditions. However, this therapeutic choice produces many side effects, restricting its use for long periods. Considering this, ethnomedicine has been proposed in the present study as an alternative treatment for menstrual problems.

Several authors have reported the existence of the modulator effects of flavonoids on the regulation of cytokines, prostaglandins and other mediators that drive immune and inflammatory reactions^13–18^. *Citrus* fruits and juices (oranges, mandarins, grapefruits, lemons, bergamots, and limes) are an important source of flavonoids, mainly glycosylflavonoids^19,20^. Among a variety of compounds present in citrus, luteolin, for example, can modulate the expression of COX-2, nitric oxide synthase, and inflammatory cytokines^21^. Hesperitin, naringenin and, rutin inhibit the COX activity as well as PGE2 production^22–24^. Naringin acts on the immune system to prevent tissue damage, while naringenin can inhibit key enzymes in the oxidation of fatty acids, as well as the NF-κβ transcription factor, reducing the production of pro-inflammatory cytokines^25,26^. Diosmin and hesperidin possess inhibitory activity over E2 and F2α prostaglandins^27^. The combination of hesperidin, nobiletin and tangeretin show a potent suppression over iNO2, TNF-α, IL-1β, and IL-6 cytokines^28^. Furthermore, in the human body, the glycosylflavonoids can be converted into their aglycone form, which has shown strong antioxidant and anti-inflammatory activities when compared to the glycosyl form^29^.

Based on this knowledge, our research group conducted a pilot study in 2014 to analyze the *citrus-therapy* effects of Tahiti lime (*Citrus latifolia)* juice (TLJ) in patients with menstrual disorders. The results showed that this *Citrus* reduced the duration and intensity of excessive bleeding, the occurrence of dysmenorrhea and the presence of clots^30^. The choice of Tahiti lime was based on preliminary empirical tests that showed the best results for *Citrus latifolia* over other limes species. In this pilot, a gynecologist used different types of lime juice during diverse menstrual cycles and identified an advantage in the activity of *C. latifolia*. This pilot also showed that the juice from one lemon fruit was capable to reduce the menstrual bleeding soon as 30 min after its consumption.

Despite the positive effects, the mechanisms that rule menstruation control are still unknown. The present research aimed to characterize by ultra-high-performance liquid chromatography,the main compounds present in the TLJ and to analyze *in vitro* and *in vivo*,the effectof TLJ in the cellular contraction and on level of prostaglandins (E2 and F2α), enzymes involved in the arachidonic acid pathway and pro-inflammatory cytokines on menstrual fluid and peripheric blood samples from women during the menstrual period.

## Results

### Characterization of Tahiti lime juice

The TLJ metabolites were identified based on fragmentation standards and the UV spectra obtained. Experiments were carried out in both negative (ESI^−^) and positive (ESI^+^) modes, varying the collision energy. Only glycosylated flavonoids were found in the TLJ. For these, the main fragment ions observed in ESI^-^ were related to sugar moiety elimination, showing the aglycone ion as the most intense. Besides, fragments from the Retro-Diels-Alder reaction and CO and CO_2_ loss were also observed^31–33^. Based on this, hesperidin, eriocitrin, rutin, and naringenin were identified in TLJ, as shown in Table 1.

**Table 1:** Glycosylated flavonoids present in Tahiti lime juice by UPLC-DAD-MS/MS (Qq-TOF).

MF		CM	t_R_	Exact mass	[Mn-H]^-^ [M + H]^+^	MS/MS^-^	Mass Error (ppm)	λmax (nm)
**Eriocitrin**	C_27_H_32_O_15_		13.7	595.1670	609.1461 611.1648	459; 287; 151	−0,2	255; 353
**Rutin**	C_27_H_30_O_16_		14.3	609.1459	609.1825 611.1647	300/301; 271; 255; 179; 151	0.4	284; 220
**Naringin**	C_27_H_32_O_14_		16.0	579.1719	595.1668 597.1829	313; 271; 151	1.8	284; 225
**Hesperidin**	C_28_H_34_O_15_		17.8	609.1819	579.1709 581.1890	301; 286; 257; 242; 151; 134	1.0	282; 213

MF: molecular formula. CM: chemical structure. t_R_: retention time.

### Effect of TLJ on apoptosis rate of C2C12 cell line

**Figure 1:**
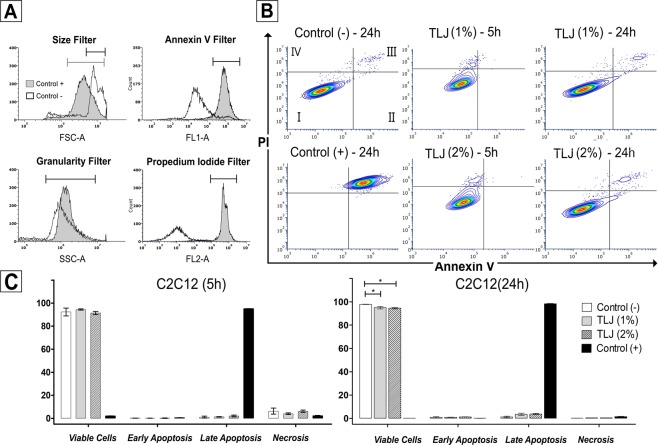
Absence of apoptotic and necrotic effects on C2C12 exposed to 1% or 2% of buffered TLJ for 5 or 24 h, analyzed by flow cytometry using AnnexinV and propidium iodide (PI) staining. (**A**) Filters used for classification of the cells; (**B**) Contour plot showing the distribution of cells classify as viable (I: PI−/Annexin V−), early apoptosis (II: PI−/Annexin V+), late apoptosis (III: PI+/Annexin V+) and necrosis (IV: PI+/Annexin V−) of the groups treated with 11 µM of H_2_O_2_ (control+), 1% or 2% of TLJ buffered for 5 or 24 h. (**C**) Bar charts showing the numeric results of the classified groups (mean ± SEM, n = 3). Statistical Analysis: ANOVA accompanied by the Dunnett’s Multiple Comparison test using the control (−) group as reference; *p < *0.05*.

**Figure 2:**
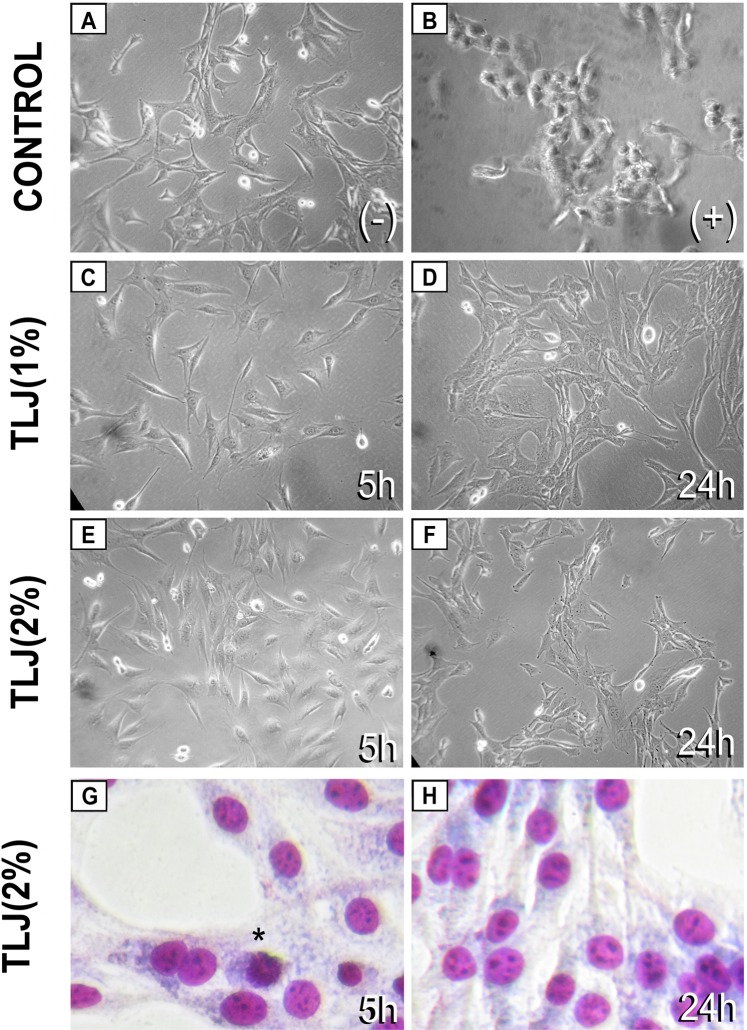
Absence of degenerative signalsof the C2C12 cells exposed to TLJ (1% or 2%) until 24 h, analyzed by phase contrast (**A–F**) or panoptic stain by bright field microscopy (**A–G**). (**A**) Control cells (5 h) without treatment showing normal morphology with some cells with signals of proliferation (small round bright cells). (**B**) Positive control cells treated with H_2_O_2_ (11 µM) at 5 h showing features of loss of viability (detachment). (**C**,**E**) cells treated at 5 h with 1% or 2% TLJ showing normal morphology (spreading) with some cells in proliferation. (**D**,**F**) cells treated for 24 h with 1% or 2% TLJ also showing normal morphology with some cells in proliferation. (**G**,**H**) micrographs of C2C12 cells stained by panoptic showing the normal characteristics in 5 and 24 h after TLJ exposition. (*) C2C12 in proliferation showing chromosome condensation.

In the experiment for apoptosis detection (FACS analysis: Annexin V - PI), there was not a significant difference in the percentage of viable cells submitted to 5 h of citrus treatment in comparison with the Control group (Fig. 1C). However, in 24 h culture, there was a small difference (<3%), statistically significant between the Control and TLJ groups that could be considered without importance in context (Fig. 1C). The citrus treatment did not induce any apoptotic or necrotic effects over myoblasts in a significant way in comparison with the Control without treatment. When analyzing the microphotographs, it was observed that the groups exposed to 1 or 2% of TLJ for 5 or 24 h showed cells with features of normality such as spreading and multiplication (Fig. 2C–F,G,H). Concerning the H_2_O_2_ treated group (Control+), the cells exposed for 5 h start to show signals of loss of viability such as detaching and rounding (Fig. 2B).

### *In vitro* Effect of TLJ on the production of PGF2α induced or not with LPS or AA

**Figure 3:**
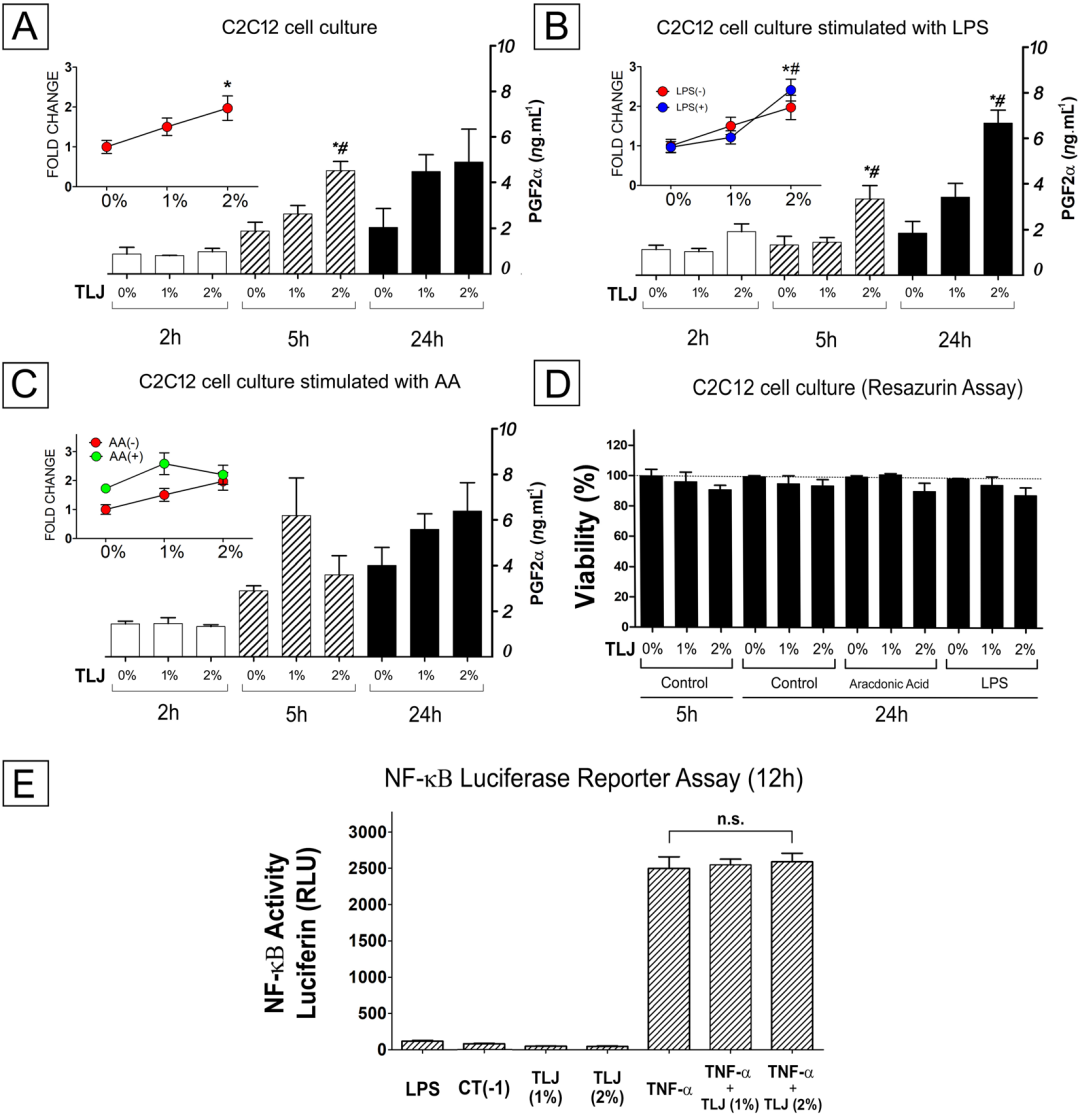
Demonstration of the augmentation of the production of PGF2α by C2C12 cells treated with TLJ without association with lost of cell viability or NF-kβ activity induction. The productions of PGF2α by the C2C12 cell line treated with different concentration of buffered TLJ (1% or 2%; pH.7.0) and exposed or not (**A**) to 10 ng of LPS (**B**) or 10 µM of Arachidonic Acid (**C**) at different time intervals (2, 5 or 24 h),were quantified by ELISA. Line graph (**A**) shows the variation of the production of PGF2α (fold change) induced by different concentrations of TLJ (n = 9; ^*^p < *0.05* in relation to the 0% Control) and bar graph shows the dynamic of the production of PGF2α (ng.mL^−1^) induced by TLJ (n = 3) at different times (*#*p* < *0.05* in relation to the 1% and 0% Controls). Line graph (**B**) shows the parity in the production of PGF2α (fold change)by cells treated by TLJ (n = 9) and stimulated or not with LPS. Bar graph shows the dynamic of the production of PGF2α (ng.mL^−1^) induced by LPS and TLJ (n = 3) treatments at different times (*#*p* < *0.01* in relation to the 1% and 0% Controls). Line graph (**C**) shows the difference in the production of PGF2α (fold change) after stimulation with arachidonic acid (AA) and treatment with TLJ (n = 9; *p* < *0.05)* in relation to the AA(−). The bar graph (**C**) shows the change of the production of PGF2α induced by TLJ (n = 3) at different times after AA stimulation. Bar graph (**D**) shows the results of the viability of the C2C12 cell line (n = 4) after 5 h or 24 h of exposition to diverse concentrations (0, 1 and 2%) of buffered TLJ quantified by resazurin assay. Bar graph (**E**) showing the activity of the NF−κβ reporter from HEK293 cells (n = 5) treated only with buffered TLJ (1 or 2%) or stimulated with TNF-α and TLJ for 12 h, in comparison with Controls group (CT-; TNF-α: 10 ng/mL and LPS: 10 ug/mL). NS: non-significant. In all the analyses, the results were represented by mean ± SEM and the ANOVA and Newman-Keuls Multiple Comparison statistic tests were used.

Comparing the results of the production of PGF2α from C2C12 cells treated with different concentrations of buffered TLJ (0, 1 and 2%) at different times (2, 5, 24 h), it was possible to see that there are positive correlations between TLJ concentration (*p* < *0.05*), the time of exposition (*p* < *0.01*) and the PGF2α level present in the supernatant (Fig. 3A). The mean level of PGF2α from the cell line exposed to 2% TLJ was significantly higher (*p* < *0.05*) than the Control and 1% TLJ, mainly after 5 h of exposition (Fig. 3A).

About the effect produced by treatment with TLJ and stimulation with LPS, the concentration of PGF2α from C2C12 exposed to citrus and LPS (Fig. 3B) were comparable in level from cells treated exclusively with TLJ (Fig. 3A). The mean level of PGF2α from the cells exposed to 2% TLJ and LPS (*p* < *0.01*) was also higher than the Control and 1% TLJ after 5 h or 24 h of stimulation. On the other hand, the TLJ treatment augmented the production of PGF2α induced by AA in an addictive way (Fig. 3C).

Analyzing the Resazurin assay results, it was possible to see that there was not a significant difference between cellular viability of the Control group to those treated with 1% or 2% of buffered TLJ for 5 h or 24 h. The viability of the myoblasts submitted to AA or LPS in combination or not with lime juice was also not different from the Control (Fig. 3D).

Concerning the NF−κβ reporter activity, HEK293 cells expressing pBIIx-luc under the control of NF−κβ increased the luciferase production in response to TNF-α after 12 h of stimulation, but when treated with TLJ (1 or 2%) did not suffer any modulation of the activity. Besides, the cells treated only with TLJ (1 or 2%) did not also stimulate significantly the NF−κβ gene reporter.

### Effect of TLJ on collagen gel contraction mediated by L929 and C2C12 cell lines

**Figure 4:**
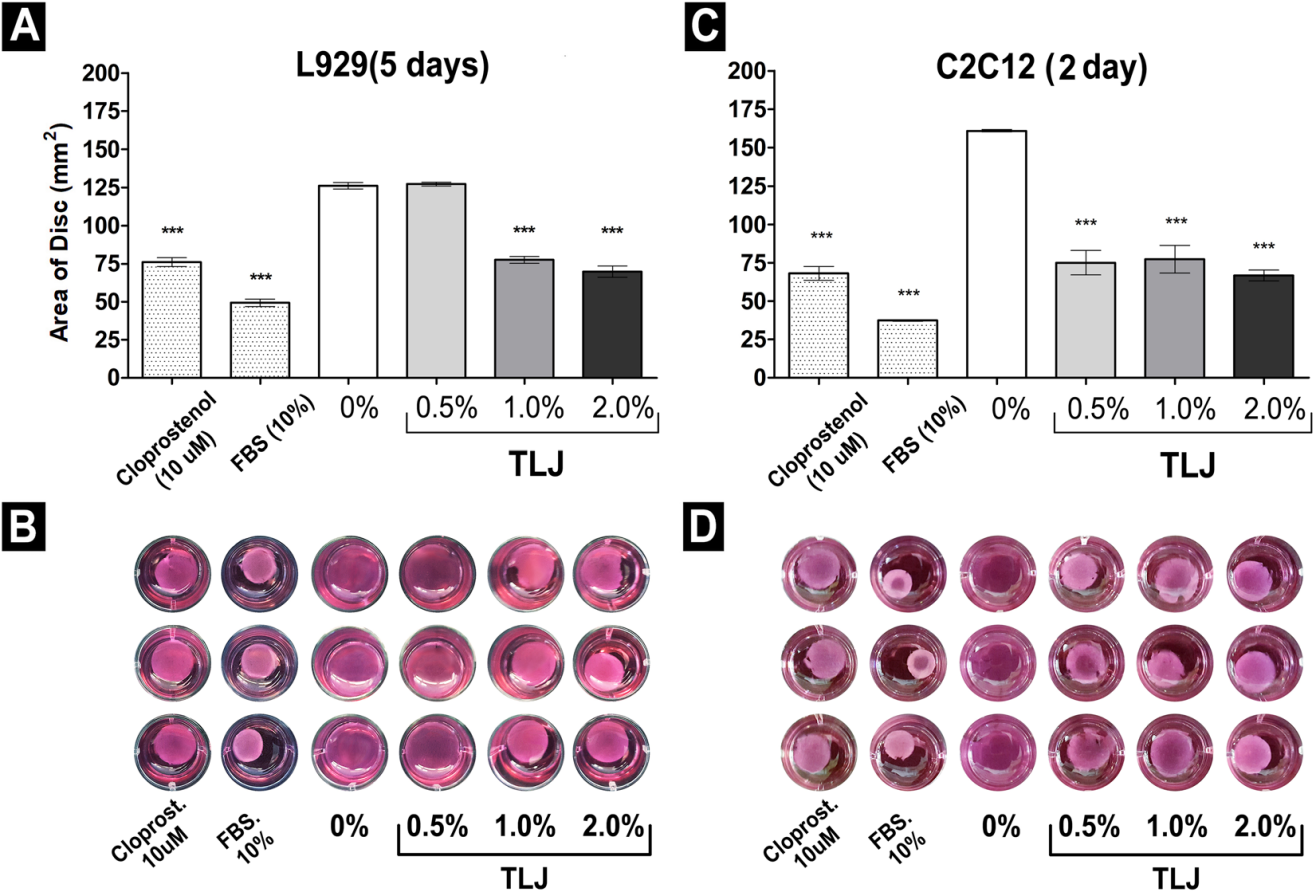
Demonstration of the contraction of collagen gel discs populated by L929 or C2C12 induced by different concentrations of TLJ. (**A**) Bar graphic comparing the mean area from collagen discs populated by the L929 cell line treated with different concentrations of buffered TLJ (0, 0.5, 1 or 2%) at 5 days in relation to the positive Controls (10 µM of cloprostenol or 10% of FBS) and negative Control (media without FBS and TLJ: 0%). (**B**) Image of the discs showing the intensity of contraction of the diverse groups after 5 days of treatment. (**C**) Bar graphic comparing the mean area from collagen discs populated by the C2C12 cell line treated with different concentration of buffered TLJ (0, 0.5, 1 or 2%) at 2 days in relation to the positive Controls (10 µM of cloprostenol or 10% of FBS) and negative Control (media without FBS and TLJ: 0%). (**B**) Image of the discs showing the intensity of contraction of the diverse groups after 2 days of treatment. Quantitative data were expressed as the mean area (mm^2^) ± SEM (n = 3). ***p < 0.001 vs. the negative Control group; Statistical analysis was performed using one-way ANOVA and the Dunnett’s post-hoc test.

The contraction assays were performed within a 24-well plate containing 1 × 10^5^/mL of L929 and C2C12 cells trapped inside of collagen gel. As shown in Fig. 4, the TJL significantly stimulated (p < 0.001) the contraction of gels with C2C12 and L929 at about 2- and 5-days post addition of treatments in comparison with the Control (Fig. 4).

### PGF2α and PGE2 levels in menstrual fluid and peripheral serum

**Figure 5:**
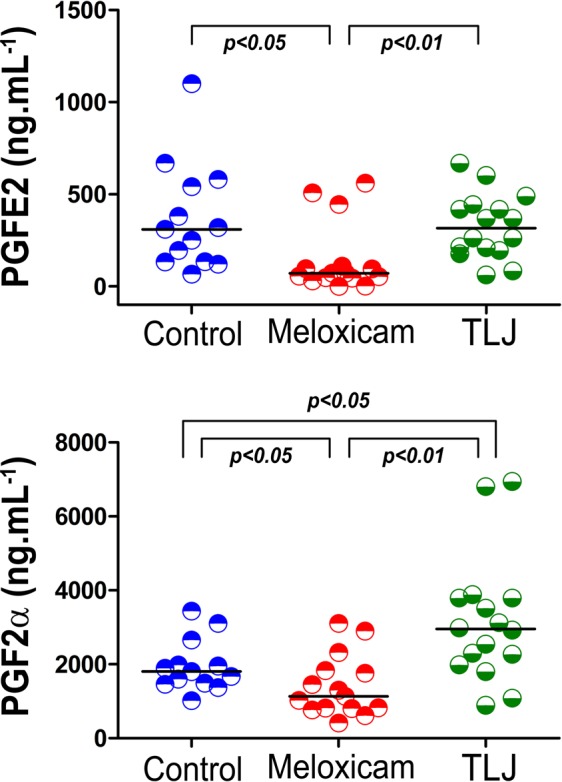
Demonstration of the augmentation of concentration of PGF2α in the menstrual fluid of women that consumed TLJ at the beginning of the menstrual phase. Scatter Plot showing the individual values and the median of the F2α and E2 Prostaglandins concentrations (ng.mL^−1^) present in the menstrual serum from the Control (n = 13), Meloxicam (n = 15) and TLJ volunteers (n = 16), collected on the second day after the beginning of the menstruation cycle. The PGE2 was analyzed by the Kruskal-Wallis and Dunn’s statistical tests for multiple comparisons and the data from PGF2α was log transformed and analyzed by ANOVA and the Newman-Keuls statistic test for multiple comparisons.

Figure 5 and Table 2 display the data relating to the concentrations of PGF2α and PGE2 in the menstrual fluid and in the peripheral serum from volunteers from the Control, Meloxicam, and TLJ groups. The results showed that the median concentrations of PGE2 from the Control (310.2 ng.mL^−1^) and TLJ (316.3 ng.mL^−1^) groups were very similar and significantly higher in comparison with the Meloxicam group (71.8 ng.mL^−1^). PGF2α values were higher in the TLJ Group (2953.1 ng.mL^−1^) than the Control (1806.3 ng.mL^−1^) and Meloxicam (1131.2 ng.mL^−1^) groups, between which the values also differed. The PGE2 concentration values from the peripheral serum samples from all groups were below the sensitivity of laboratory testing. However, in terms of PGF2α measurements, the Meloxicam group had a significantly lower value (1.32 ng.mL^−1^) than the TLJ (2.44 ng.mL^−1^) and Control (3.16 ng.mL^−1^) groups. There was not any participant that had harm or unintended effects in the groups.

**Table 2:** Comparison of the concentrations of the prostaglandins E2 and F2α present in the menstrual fluid and the peripheral serum from volunteers from the TLJ, Meloxicam and Control groups.

PG		GROUP	N	MEAN	SEM	MEDIAN	P25	P75
**Menstrual Fluid**	**PGE2 (ng.mL**^**−1**^**)**	TLJ (a)	16	328.3	43.6	316.3	199.1	438.3
		Meloxicam (b)	15	146.3	48.9	71.8	44.9	109.5
		Control (a)	13	369.4	80.6	310.2	133.3	561.7
	**PGF2α (ng.mL**^**−1**^**)**	TLJ (a)	16	3165.1	426.9	2953.1	2057.0	3789.3
		Meloxicam(b)	15	1402.3	213.3	1131.2	801.8	1827.2
		Control (c)	13	1955.1	194.8	1806.3	1474.5	2315.0
**Peripheral Blood**	**PGF2α (ng.mL**^**−1**^**)**	TLJ (a)	16	3.37	1.05	2.44	1.34	3.32
		Meloxicam (b)	15	1.86	0.37	1.32	0.87	2.59
		Control (a)	13	5.25	1.21	3.16	1.42	10.65

Legend: SEM - Standard Error Mean, P25 - 25% percentil, P75 - 75% percentile, The letters (a), (b) and (c) represent groups considered statistically different. Statistical tests: Kruskal-Wallis and Dunn for multiple comparisons. PGE2 values from all peripheral blood samples were below the sensibility of the ELISA kit.

**Figure 6:**
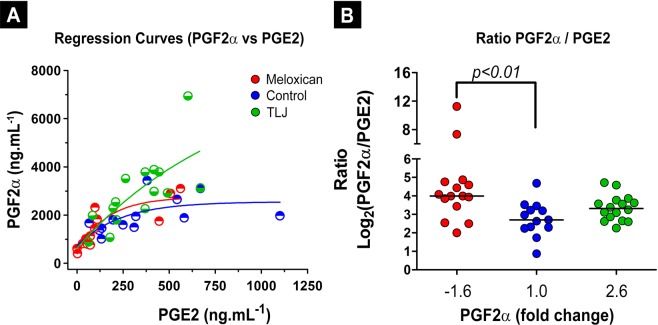
Demonstration of the augmentation of concentration of PGF2α without alteration of PGE2 in the menstrual fluid of women that consumed TLJ at the beginning of the menstrual phase in contrast with those were treated with Meloxicam. (**A**) Best fitting curve (PGF2α vs PGE2) drawn from PGs concentrations quantified from menstrual fluid collected on the second day after the beginning of the menstrual cycle, from the Control (n = 13), Meloxicam (n = 15) and TLJ (n = 16) volunteer groups. (**B**) Scatter graphic showing the individual values and medians of the ratio (PGF2α/PGE2) in relation to change fold of PGF2α of the Control, Meloxicam and TLJ groups. The medians were compared using the Kruskal-Wallis and Dunn´s statistical tests for multiple comparisons.

Figure 6 shows the dispersion curves formed by combining the concentration values of the PGE2 and PGF2α of the menstrual fluid of each volunteer from the three groups. The PGF2α values in the TLJ group increased more intensely than the PGE2 values, causing an increase in the slope of the curve of the group in comparison with the Control and Meloxicam groups. In the Meloxicam group, there were reductions of PGE2 and PGF2α, which maintained the slope of the Meloxicam curve equivalent to that of the Control group. This behavior can be observed in the scatter plot (6B) which shows only a difference in the median of the ratio (PGF2α/PGE2) between the Control and Meloxicam groups (*p* < *0.01*) independent of the level of PGF2α.

### TNF-α, IL-1β and IL-6 cytokines in menstrual fluid and peripheral serum

**Figure 7:**
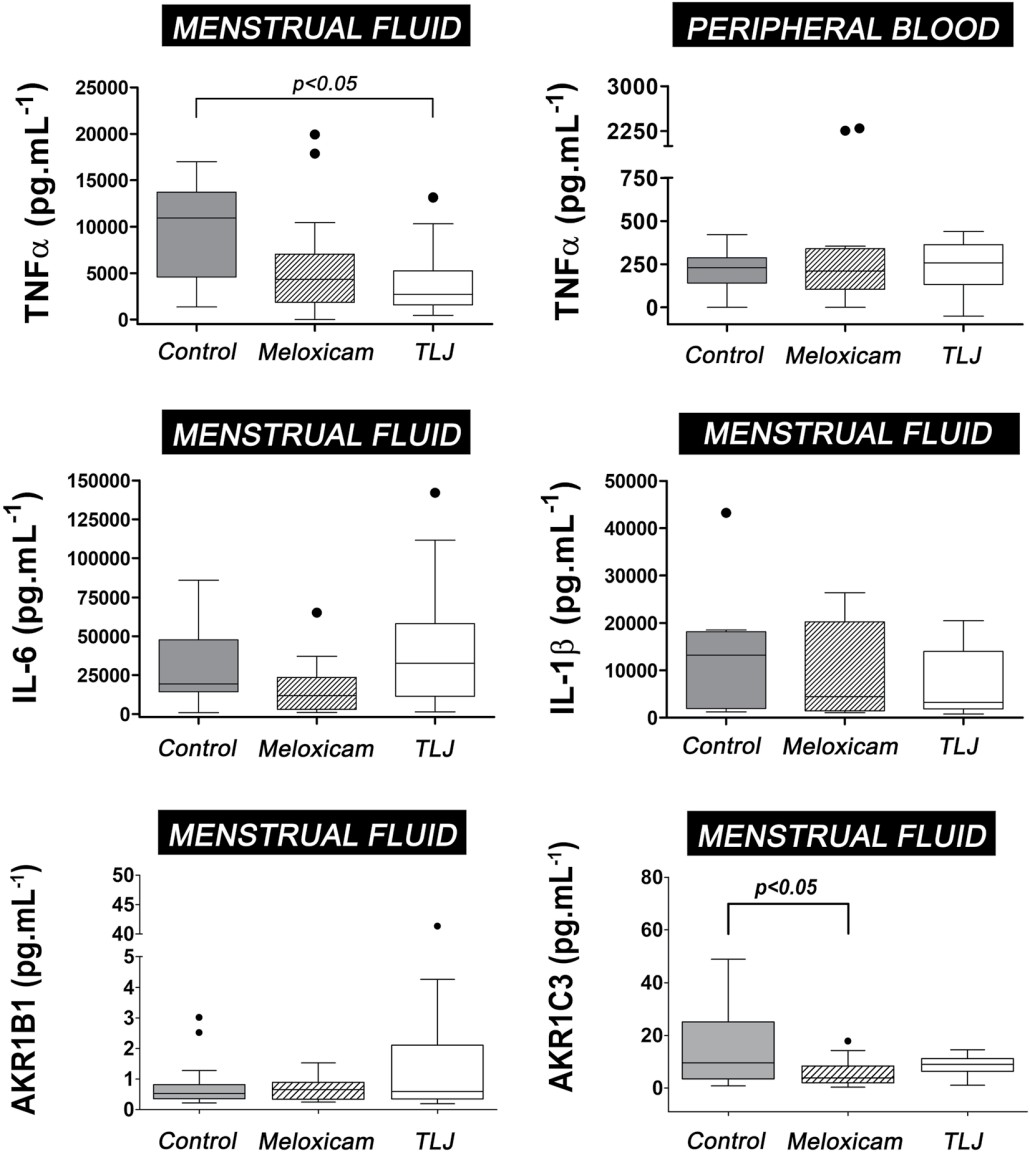
Reduction of the menstrual level of TNF-α from women that consumed TLJ at beginning of the menstruation. Box-plot showing the comparing of the levels of TNF-α, IL-6, IL-1β and of the enzymes AKR1B1 and AKR1C3 from menstrual fluid or peripheral serum of women submitted or not (n = 14) to TLJ (n = 16) or meloxicam (n = 16). The medians were compared using the Kruskal-Wallis and Dunn´s statistical tests for multiple comparisons.

The Fig. 7 shows the statistical analysis of the TNF-α, IL-1β and IL-6 cytokines present in the menstrual fluid and peripheral serum of the volunteers of the Control, Meloxicam, and TLJ groups. In terms of the concentrations of TNF-α in the menstrual fluid, the group that used the TLJ had a significantly lower median value (2717 pg.mL^−1^) than the Control group (10950 pg.mL^−1^). However, in comparison with the Meloxicam group (4335 pg.mL^−1^), there was no statistical difference. In relation to the IL-1β and IL-6 cytokines, no difference in the median concentrations was observed between the groups analyzed. Concerning the TNF-α cytokine concentration present in the peripheral serum, the results showed that there was no difference in median cytokine levels between the TLJ (258.4 pg.mL^−1^), Meloxicam (210.3 pg.mL^−1^), and Control (229.6 pg.mL^−1^) groups. The IL-6 and IL-β values measured were below the quantification sensitivity of the ELISA kits and were therefore not computed in the analysis.

### AKR1B1 and AKR1C3 in menstrual fluid and peripheral serum

Figure 7 shows the statistical analysis of the concentration of the AKR1B1 and AKR1C3 present in the menstrual fluid from the Control, Meloxicam and TLJ group volunteers. It was observed that the median concentration of AKR1B1 in the menstrual fluid did not differ in any of the three groups (Control: 0.5 pg.mL^−1^; Meloxicam: 0.7 pg.mL^−1^; TLJ: 0.6 pg.mL^−1^) . On the other hand, the treatment with the Meloxicam induced a significant reduction in the concentration of AKR1C3 in comparison with the Control group  (Control: 9.6 pg.mL^−1^, Meloxicam: 3.9 pg.mL^−1^; TLJ: 9.1 pg.mL^−1^). Concerning the serological level of AKR1C3, there were no differences between the three groups (Control: 3.4 pg.mL^−1^; Meloxicam: 2.5 pg.mL^−1^; TLJ: 3.9 pg.mL^−1^). The peripheric blood AKR1B1 levels from all groups were below the sensitivity of ELISA kit and therefore it was not computed in the analysis.

## Discussion

**Figure 8:**
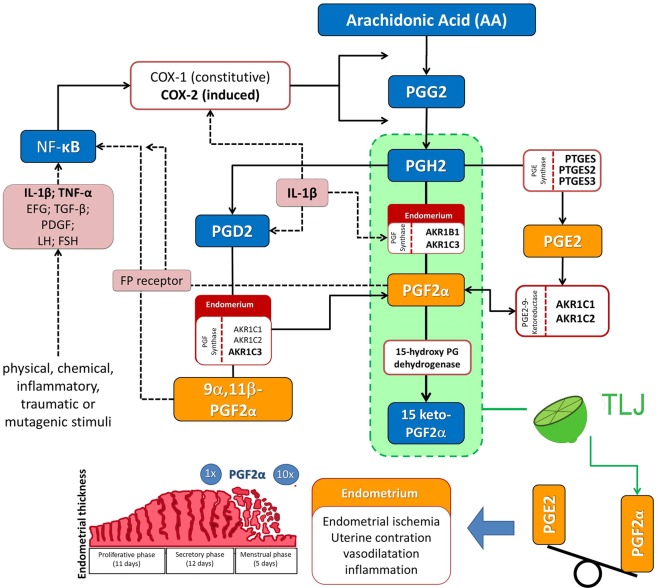
Pathways responsible for producing E2 and F2α prostaglandins. PGF2α and PGE2 are produced from arachidonic acid (AA) by cyclooxygenase enzymes (COX-1 and COX-2). In the first step, AA is metabolized in PGG2 intermediate by cyclooxygenase and, sequentially, in PGH2 by peroxidase. The PGH2 can be synthesized directly into PGF2α by the AKR1B1 or AKR1C3 enzymes. Both AKRs are present in the endometrium during the menstrual cycle: AKR1B1 is in the glandular epithelium and stromal cells, and AKR1C3 is situated in the epithelial cells. PGH2 can also be converted into 9α,11β-PGF2α by the AKR1C3 and to a lesser extent by the AKR1C1 and AKR1C2 enzymes. The 9α,11β-PGF2α is a stereoisomer of PGF2α and has the same potency in the contraction of smooth muscle fibers. Both the AKRs and F2α prostaglandins (PGF2α and 9β, 11β-PGF2α) act on the transcription of the NF−κβ factor, inducing the production of COX-2. Furthermore, the AKR1B1 and AKR1C3 activities in the endometrium can be stimulated by pro-inflammatory cytokines, which can lead to a vicious inflammatory cycle. Finally, PGH2 can be metabolized into PGE2 by the PGE synthases (PGES, PGES2 or PGES3) followed by conversion into PGF2α by PGE2,9-ketoreductase (AKR1C1 and AKR1C2)^35–38^.

The human endometrium is a dynamic tissue, whose function is mainly regulated by the activities of the estrogen and progesterone hormones. During menstruation, many of the events that affect the endometrium tissue are inflammatory in nature. Among several mediators, prostaglandins stand out as important inflammation agents due to their activity in the control of the uterine musculature contraction, caliber, and vascular permeability. Several studies have associated disorders of the menstrual cycle, such as excessive menstrual flow and dysmenorrhea with abnormalities in the production of prostaglandins, especially E2 and F2α^3,4,7,34^ (Fig. 8).

The discovery that certain *in vitro* flavonoids are selective modulators of prostaglandin has led to speculation that these compounds, which are present in citrus fruits, could be primarily responsible for an anti-inflammatory mechanism. In our analysis, *Citrus latifolia* exhibited some flavonoids that are typically found in other limes, such as hesperidin, eriocitrin, rutin and naringin (Table 1). Some authors have found the hesperidin, diosmin, and eriocitrin flavonoids in different citrus juices^39,40^. Another study found hesperidin to be the main component of the Tahiti lime, followed by eriocitrin, rutin, naringenin, narirutin and diosmin^41^. In relation to its biological features, hesperidin and eriocitrin metabolites have antioxidant and anti-inflammatory activity capable of eliminating free radicals and inhibiting inflammation *in vitro*. Hesperidin significantly decreased the production of PGE2 without altering the COX-2 level. Rutin is capable of inhibiting IL-1β cytokine production, apoptosis and edema, reducing the inflammatory response^42–45^.

In the present study, the *in vitro* level of PGF2α and PGE2 produced by murine myoblast cells (C2C12) when exposed at different concentrations of TLJ (1% and 2%) and times (2, 5 , and 24 h) were measured (Fig. 3). The PGE2 concentration was not enough to be detected in our analyzes. The PGF2α level at both 1 and 2% TLJ concentrations, mainly at 5 h exposure time, was significantly higher compared to the Control (0% TLJ) (Fig. 3A). In relation to experiments of co-stimulation, the results showed that there was no interaction between TLJ and LPS stimulation on the production of PGF2α indicating that the action mechanism of TLJ may be independent of the production of pro-inflammatory mediators (Fig. 3B). However, the co-stimulation TLJ/AA showed an additive interaction that was responsible for the augmentation of the level of PGF2α, indicating that the TLJ could act directly over the main branch of the AA pathway (Fig. 3C). In endometrial tissue, prostaglandins are synthesized from the cell membrane phospholipid precursor, which is undergoing the death process (Fig. 8). In our case, the results of Figs. 1 and 2 showed that the viability of C2C12 when exposed up to 2% of TLJ at 24 h was not affected (Figs.1, 2 and 3D), guaranteeing the cellular integrity and discarding the possibility that PGF2α production was due to any cell destruction caused by TLJ exposure.

One of the proposed mechanisms that would explain the control of menstruation using citrus would be the possibility of stimulating smooth muscle contraction. Up to this point, we determined that PGF2α production could be equally stimulated by TLJ *in vitro* as *in vivo*. But we do not know whether such stimulation could induce effective contraction. To respond to this challenge, we performed a collagen contraction assay using two cell lines (C2C12 and L929) comparing various concentrations of TLJ with the synthetic PGF2α and SFB (positive Controls), known to stimulate contraction^46^. The results showed that the TLJ induced a contraction of the collagen disc, mainly in a concentration above 1%. In relation to cell lines, the contraction of myoblast (C2C12) was more intense and faster (5 days vs2 days) than fibroblast lineage (L929). These data may explain the relationship between lime juice consumption and decreased menstrual flow since PGF2α could age in the contraction of both the blood vessel and myometrium.

The stimulatory assay using the myoblast cell showed the ability of TLJ to induce PGF2α production *in vitro*, but the question over its activity in the uterus remained open. To address this issue, during the menstrual period, we compared the production of PGs, enzymes and inflammatory mediators associated with the AA pathway, using menstrual fluid and peripheric blood samples from women submitted to TLJ or non-steroidal anti-inflammatory drugs (NSAID); or without any intervention. The results showed that the concentration of PGE2 in the menstrual fluid of the volunteers from the Control and of the TLJ treated groups remained practically equivalent (Table 2). However, in the group treated with Meloxicam, the PGE2 concentration was lower than in the other groups, although the volunteers did not present any type of menstrual disorder (Table 2). In contrast, the expressive increase in PGF2α levels was found in the menstrual serum of the women from the TLJ in comparison with the participants from the Control or Meloxicam groups (Fig. 5). These results were corroborated by another work of our group, whose objective was to evaluate a methodology based on SPE-LC-MS/MS for the simultaneous quantification of prostaglandins (PGE2 and PGF2α) from the menstrual fluid. The experiments were carried out using independent samples in relation to present study: group Control (n = 15) and a group of women exposed to TLJ (n = 7). The PGF2α levels were also found to be higher in the treated group, reinforcing an effect found previously of the intake of TLJ^47^.

Regarding the regression analysis, the results of the relationship between the PGF2α and PGE2 (Fig. 6A,B) revealed that the slope of the best adjust curve was more accentuated in the TLJ than the other two groups. This result is mainly related to the increased production of PGF2α in comparison with the PGE2 in the TLJ group. On the other hand, the individual concentrations of PGF2α and PGE2 in the Meloxicam group were generally lower than in the other two groups which are in accordance with the literature^48^. The slope of the PGF2α/PGE2 curve of the Meloxicam group followed the control pattern.

The *in vitro* and *in vivo* data indicated that TLJ may exert a regulation of menstrual flow, inducing an increase of uterine PGF2α and maintaining the PGE2 level unchanged. PGF2α produced by citrus therapy may increase the capillary resistance acting on prostaglandins receptors^49^, reducing the menstrual flow. Studies showed that naringin, rutin, eriocitrin and hesperidin, four of the found compounds of TLJ were unable to inhibit the PGE2 production induced by the LPS *in vitro*^50^. This effect could benefit women suffering from menstrual disorders associated with lower production of PGF2α in a superior manner in comparison with the treatment based on the use of NSAIDs. Anti-inflammatory drugs concomitantly inhibited PGE2 (menstrual) and PGF2α (peripheral and menstrual) production, which could lead to an increase in the incidence of side effects, since PGE2 has a significant physiological role in the uterus and other organs^51^.

Analyzing the endometrial concentration of PGs from normal women or those with menorrhagia, Smith *et al*. (1981) proposed that excessive blood loss may be related to the possible conversion of PGF2α to PGE2^6,52^. The exact mechanism leading to increased production of PGF2α by TLJ remains unknown. In the present study, treatment with TLJ did not significantly affect the concentration of AKR1B1 and AKR1C3 (serological or menstrual) in comparison with the Control group. The Meloxicam-treated group experienced a significant decrease in menstrual AK1C3. As a result, the increase in the slope of the PGF2α/PGE2 curve in the menstrual blood of the TLJ-treated volunteers is likely to be unrelated to a change in the concentration of the AKR1B1 and AKR1C3 enzymes.

During inflammatory events, there is an increase in the production of cytokines, mainly TNF-α and IL-1β, which have important functions in maintaining the pro-inflammatory uterine profile. The IL-1β cytokine can stimulate the  NF−κβ transcription factor, increasing the expression of genes related to COX-1 and COX-2. In a pro-inflammatory situation, as occurs in the menstrual process, we could expect greater production of COX-2, which would potentiate the production of PGs. Several studies have shown that flavonoids modulate AA metabolism through the inhibition of COX-2, PLA2 and nitric oxide, producing enzymes (iNOS), which consequently inhibit the synthesis of eicosanoids and prostaglandins^53^.

Monitoring of NF−κβ activation after TLJ treatment could help to a better understanding of the mechanism of action of the citrus. In our case, the data showed that the exposition to TLJ did not contribute to inhibit or stimulate the NF−κβ signaling (Fig. 3E). We speculated that the direct modulation of NF−κβ/COX2 activity may not be the main mechanism responsible for the control of menstrual dysfunction mediated by TLJ. The results of the present study showed that the concentration of PGE2 in the menstrual blood did not change to the Control group. The concentrations of IL-1β and IL-6 did not differ significantly among the three groups. Only the menstrual TNF-α concentration showed a decrease in the TLJ and Meloxicam groups in comparison with the Control group (Fig. 7). The *in vitro* experiments with NF−κβ reporter showed the TLJ treatment was not capable to inhibit or stimulated the transcription of the reporter gene. Besides, if inhibition of NF-kβ/COX-2 activity had occurred, as reported in the *in vitro* studies, likely lower values of the *in vitro (PGF2α)* and *in vivo (PGF2*α, IL-1β, and IL-6) pro-inflammatory mediators analyzed would have been observed in the TLJ group. However, specific biochemical studies are needed to assess the effects of the treatment with TLJ in the arachidonic acid pathway.

Unlike most studies that use the epicarp to obtain the essential oils or pure flavonoids in high concentrations, we used juice extracted from the endocarp as a therapeutic component. This specificity may explain the differences in the results related to the inhibition of COX and pro-inflammatory cytokines found *in vitro* studies. The composition of edible and inedible parts of citrus are different and the flavonoids present in the edible part are more beneficial than those in the peel^39^. The literature shows that the chemical structure of the flavonoidsis associated with the capacity of COX2 activity inhibition. A study showed that the 50 µM of hesperidin (a major compound of TLJ), in contrast with other types of flavonoids absent in the TLJ (quercetin, luteolin, disometin, genistein..), does not change the expression of COX2 and inhibits the IκB-α phosphorylation and NF-κβ p50 and p65 nuclear translocation stimulated by LPS^54^. Eriocitrin, another abundant flavonoid of TLJ, in concentration of 100 µM also showed a lower capacity of COX2 inhibition in contrast with the other group of flavonoids often absent in TLJ^55^.

Besides, the inhibitory activity of flavonoids is dose-dependent. LPS-induced gene expression of COX-2 was inhibited by hesperidin at a concentration superior to 250 µM^56^. Studies in animals often use a concentration of hesperidin around 100 mg/kg/day^57^. In a human clinical trial, volunteers consumed two capsules of 146 mg of hesperidin daily (~50 µM of serum level, considering the blood volume of 5 L) for 4 weeks and the leukocyte cytokine production after PHA stimulation was analyzed. Results showed that hesperidin consumptions do not induce immunomodulation of basal immune cell functions and their activation capacities^58^. In our case, the volunteers consumed 1 lime (40 ml of TLJ)/ day, which contains ~20 mg of hesperidin (http://phenol-explorer.eu/contents/polyphenol/207).

At this point, we can show the intake of the lime juice induces the production of the PGF2α uterine, which is probably responsible for decreasing of the menstrual bleeding. In the TLJ exist compounds of unknown classes, that individually or in combination can stimulate *in vitro* and *in vivo* the cascade of the arachidonic acid and other pro-inflammatory pathways. In the pilot study, the results showed that in general, volunteers with increased menstrual flow who underwent citrus therapy benefited from the treatment. Some of the volunteers exhibited a reduction in just days (23%), bleeding (72%) and clot reduction (43%) while some had complete remission of dysmenorrhea (21%)^30^. The modulation of the prostaglandin production provided by citrus-therapy, probably induced a reduction of the vessel caliber and menstrual flow, without potentiating the uterine contractions related to painful symptomatology. It may be that the increase in PGF2α was not enough to increase contractions of the myometrium and intensify cramps during menstruation. Moreover, it is likely the decrease in cytokines production, such as TNF-α, has contributed with the inhibition of the inflammatory process and nociceptive activities. Corroborating with the findings of the present study, other authors showed that the treatment with Rosemary (*Rosmarinus officials L.)*, a medicinal plant involved in modulation of prostaglandins from the *Lamiaceae* family, also reduces the amount of menstrual bleeding and dysmenorrhea similar to mefenamic acid^59^. Hesperidin and eriocitrin (eriodictyol), present in the TLJ, were found between the 57 (poly) phenolic profiles identified in the Rosemary^60^.

Based on the results found *in vitro* and *in vivo*, we propose that the components present in the Tahiti lime endocarp facilitated action in the first route of PG synthesis, since the increase of PGF2α in the menstrual flow does not seem to be accompanied by alterations in the PGE2 concentration. The effects of citrus therapy shown by our results were restricted to the menstrual fluid and did not have any systemic repercussion. During menstruation, the desquamation process produces a high level of inflammatory mediators, and It is unlikely that the TLJ could  potentialize a rise. We can speculate that the TLJ action is more related to modulation of the PGF2α metabolization than its own production.

## Conclusion

Glycosylated flavonoids present in the Tahiti lime juice appear to act in the PGF2α uterine pathway, favoring the control of menstruation mediate by a muscular contraction.

## Methods

### Characterization molecular of Tahiti lime juice

#### Chemicals and reagents

All organic solvents were HPLC grade and purchased from Mallinckrodt Baker (St. Louis, MO, USA). The water used for the mobile phase preparation was purified by a Milli-Q purification system (Millipore, São Paulo, Brazil). The formic acid (≥95%) was purchased from Sigma-Aldrich (St. Louis, MO, USA). All other reagents were of analytical grade. Flavonoid and organic acids standards, hesperidin (hesperetin 7-O-rutinoside), eriocitrin (eriodictyol 7-O-rutinoside), narirutin (naringenin 7-O-rutinoside), naringin (naringenin 7-O-neohesperidoside), rutin (quercetin 3-O-rutinoside), naringenin (4′,5,7-trihydroxyflavanone), quercetin (3,5,7,3′, 4′-pentahydroxyflavone), caffeic acid (3,4-dihydroxycinnamic acid) and gallic acid (3,4,5-trihydroxybenzoic acid) (≥97.0%) were all HPLC grade and purchased from Sigma-Aldrich. The standard and stock solutions were stored at −20 °C during the analyses. The mobile phases were prepared in a volume/volume ratio.

#### LC–MS system

The ultra-high performance liquid chromatographic (UHPLC) system (Shimadzu Kyoto, Japan) consists of two LC-30AD quaternary pumps, both equipped with a low-pressure gradient unit, a SIL-30AC self-injector with a 500 μL sampling loop, two DGU-20ASR degassers, a CTO-20AC oven, and a CBM 20A controller; coupled to a DAD detector (SPD-M30A) and a QqToF Impact HD mass spectrometer (BrukerDaltonics GmbH, Germany) equipped with an electrospray ionization (ESI) source. Total and production (auto-MS/MS) experiments were carried out in the two ionization modes: positive and negative, within a mass range of m/z 80 to 1300. The ionization condition sources were nebulizer 1.7 bar, drying gas flow 9.3 L.min^−1^, drying temperature 200 °C, end plate and capillary voltage of 500 V and 4500 V, respectively. The analyses were performed in reverse elution mode using the Waters XSelect HSST3 column (3.5 μm, 2.1 × 100 mm).

### Sample preparation: Tahiti lime juice and standards solutions

The fruits were purchased from local retail outlets and washed. The juice of five limes was hand-squeezed and mixed. It was then centrifuged for 10 min at 8000 g and 20 °C. The supernatant was submitted to off-line Solid Phase Extraction (SPE) using OASIS-HLB cartridges. Cleanup was performed with H_2_O (0.1% formic acid) and the analytes were eluted with ACN:MeOH (50:50 v/v) (organic fraction). The organic fraction was evaporated in a Speedvac, 40 °C, for 12 hours. Subsequently, 15 mg of the organic fraction was re-suspended in 1 mL of ACN:H_2_O (5:95 v/v, 0.1% formic acid) and injected into the chromatographic system (15 mg.mL^−1^). The standard solutions were prepared in ACN:H_2_O (5:95 v/v, 0.1% formic acid) (1 μg.mL^−1^).

### *In vitro* effect of tahiti lime juice on the production of prostaglandins and in the cellular contraction

#### Cells culture

Immortalized mouse fibroblast (L929) and myoblast (C2C12) cells were cultured at 37 °C and 5% CO_2_ in DMEM-high glicose medium supplemented with L-glutamin 2 mM, 10% FBS (Cultilab, São Paulo, Brazil) and 1% antibiotics Pen/Strep 10,000 U (Vitrocell-Embriolife, Campinas, SP, Brazil). Adherent cells were harvested with 0.25% trypsin (Thermo Scientific) and viable cells were determined by the trypan blue exclusion method. The experiments were only carried out with 90% of cell viability.

### Preparation of TLJ for cell culture treatment

Tahiti lime fruit was washed, cut, squeezed, centrifuged at 1000 G and filtered using a sterile syringe filter with a 0.22 µm pore size (MillexSyringer-driven Filter PVDF – Millipore, Cork, Ireland). Subsequently, the filtered juice was diluted with sterile deionized H_2_O at 10% concentration and the pH of the solution was adjusted using NaOH (1 M). The buffered citrus solution was used to produce completed DMEM media with the lime juice at final work concentrations of 1.0 and 2%.

### Effect of Tahiti lime juice on viability of C2C12 analyzed by resazurin

The viability of the C2C12 cells treated for 5 and 24 h with 1 or 2% of TLJ were determined by Resazurin assay as described previously^61^. C2C12 at concentrations of 1 × 10^5^ cells.ml ^−1^ was seeded in a 96-well (Corning Incorporated, NY, USA) and incubated in quadruplicate. At the end of the exposures periods and after washing of wells with PBS, 200 ul of DMEM culture media containing Resazurin (70 µM final concentration – Sigma-Aldrich, cat. R7017) was added to each well. The plates were gently shaken and incubated for 4 h at 37 °C in 5% CO_2_ atmosphere. By monitoring the absorbance at 570 nm and 600 nm using the UVM340 monochromator, the relative viability of the groups was determined using as reference, the cells incubated only with media or DMEM with resazurin without metabolization.

### Apoptotic Effects of TLJ on C2C12 myoblast cell line

For analysis of apoptosis, C2C12 cells (1 × 10^6^ cells.mL^−1^) were cultivated in triplicate in 6 wells plate (Corning Incorporated, NY, USA). After 5 h and 24 h of treatment with 1 or 2% of TLJ, detached and adherent cells, harvested with ethylenediaminetetra-acetic acid (EDTA, Sigma-Aldrich), were collected in the same tube, washed with PBS and centrifuged at 900 G for 5 min. After discarding the supernatant, the pelleted cells were re-suspended in 400 µL binding buffer. An aliquot of suspension (200 ul) was transferred to tubes and stained with 2.5 µL of Annexin V and 5ul of propidium iodide (PI) solutions (APOAF, Sigma-Aldrich, São Paulo, Brazil). The tubes were incubated for 10 min at room temperature in the dark and the cell suspensions were featured by flow cytometry (BD Accuri™ C6 Plus Flow, San Jose, CA, USA)^62^. The distribution of cells classified as viable (PI−/Annexin V−), early apoptosis (PI−/Annexin V+), late apoptosis (PI+/Annexin V+) and necrosis (PI+/Annexin V−) was performed by the Express 6 Plus software (De Novo, Glendale, CA, USA) using as controls, cells grown only in completed DMEM (control−) or treated with 11 µM of H_2_O_2_ (control+)^62^.

### Effect of TLJ on the production of PGF2α by myoblasts co-stimulated or not with LPS or arachidonic acid by myoblast

The modulatory effect of TLJ on the production of PGF2α was analyzed, stimulating C2C12 with LPS (Sigma: *Escherichia coli* O111:B4) or arachidonic acid (AA) (Sigma: A3611), using the non-exposed cell as a control. Next, C2C12 cells, at a concentration of 10^5^ cells.well^−1^ (96 well-microplate - Corning Incorporated, NY, USA), were cultivated for 24 h in DMEM media supplemented with 10% of SFB (serum fetal bovine) and antibiotics (penicillin/streptomycin), using an incubator at 5% of CO2 at 37 °C. After this period, the C2C12 were previously (30 min) treated with different concentrations of 1 or 2% of TLJ prepared just before use and co-stimulated or not with LPS (10 ng.mL^−1^) or AA (10 µM) at different times (2, 5 and 24 h). PGF2α values from the supernatant of the C2C12 cultures were quantified using ADI-901-069 ELISA kit (EnzoLife Sciences, Farmingdale, NY, USA).

### Effect of TLJ on *In vitro* monitoring of NF-kB activation and inhibition

HEK293 cells expressing pBIIx-luc^63^, were cultivated in 96-well Corning Costar^®^ plates at 5 × 10^5^ cells.well^−1^. After 24 h, the cells were treated with TNF-α 10 ng.mL^−1^ (Sigma), TJL (1% and 2%), TJL (1% and 2%) + TNF-α 10 ng.mL^−1^, LPS 10 µg.mL^−1^ (Sigma), in serum deprived DMEM without phenol red (Gibco, Carlsbad, CA, USA) by 12 h. Cells were lysed by Dual-Glo Luciferase assay kit (Promega, Madison, WI, USA), transferred to white plate 96-well Corning Costar^®^ plate and Firefly and Renilla luciferase activity measurement were obtained in a SpectraMax i3 luminometer (Molecular Devices, Sunnyvale, CA, USA).

### Effect of TLJ on collagen gel contraction mediated by L929 and C2C12 cell lines

Tubes with 400 μL of DMEM medium, 1 × 10^5^ cells.mL^−1^ (L929 or C2C12) and 200 μL of collagen solution (3 mg.mL^−1^ in 0.1% acetic acid) (ChemCruz, Dallas, Texas, USA: SC136157) were prepared. After the addition of collagen, 4 μL of 1 M NaOH was immediately used for activation of the polymerization. The volume of 500 μL of the mixture was transferred to each well of a 24-well plate and the gel was left for 20 minutes at room temperature until its solidification. Next, 600 μL of DMEM medium without FBS was added to each well and the gel was dissociated from the well by gently running the tip of a 200-μL pipet tip along the gel edges without shearing or tearing the gels. The plate was placed into an incubator at 37 °C and 5% CO_2_ overnight. After this time, the supernatant was removed and fresh DMEM medium with *Citrus latifolia* juice was added to the culture at three different concentrations (0.5%, 1%, and 2%), in triplicate. Cells cultivated in medium with FBS were considered as a positive control, as well as cells cultivated in medium with 10 μM of a synthetic PGF2α, Cloprostenol Sodium (Cioprostinn, Vetecia Lab, Jacareí, SP, Brazil). Cells cultivated in the absence of FBS were considered as a negative control. The contraction of gels with L929 and C2C12 cells were observed in several time-points (0 h, 12 h, 24 h, 48 h, 96 h, and 120 h) and the diameter changes of collagen disks were recorded using a digital camera at a fixed distance in order to obtain images at each time-point.

### *In Vivo*: - effect of Tahiti lime juice on level of prostaglandins (E2 and F2α), enzymes involved in the arachidonic acid pathway,and pro-inflammatory cytokines on menstrual fluid and peripheric blood samples during menstruation period

#### Selection of volunteer participants

Forty-five volunteers aged 18 to 40 years from the city of São Carlos (São Paulo State, Brazil) were enrolled in the present study after agreeing and signing the informed consent form. The Research Ethics Committee (CEP) of the Federal University of São Carlos (n° 48233715.8.0000.5504) specifically approved the present study. The inclusion and exclusion participation criteria were women that did not use any type of hormonal contraceptive, with no history of gynecological diseases, immunodeficiency, autoimmune diseases, malignant neoplasms or history of chemotherapy or radiotherapy.

The G1 group (TLJ group; n = 15) was instructed to ingest one dose of Tahiti Lime juice (~20–30 mL) diluted in water: at the beginning of bleeding (30 min before meals) and on the second day of menstruation in the morning. The G2 group (Meloxicam group; n = 15) received two tablets of 15 mg of meloxicam drug (Meloxicam®, Novamed - ProdutosFarmacêuticosLtda, Manaus, Amazonas, Brazil) and took the medication in the same manner as the TLJ: at the beginning of bleeding and another on the second day of menstruation. The G3 group (Control group; n = 15) was instructed not to use any type of medication and not to consume any type of citrus fruits in the first two days of menstruation. Most of the prostaglandin release occurs during the first 48 h of the menstruation, which corresponds to the period of greatest exacerbation of the symptoms^64^.

### Registration number and name of the clinical trial

The clinical data from the present work was deposed in a Clinical Trial Database and referred to*Influence of lemon on the inflammatory activity of healthy women during menstruation* (number: ReBeC - RBR-3tknxy), registered in REBEC (http://www.ensaiosclinicos.gov.br/).

### Peripheral blood and menstrual fluid

Peripheral blood samples were collected using non-anticoagulant vacuum tubes (Vacutainer Biosciences, Franklin Lakes, NJ, USA). After being left to stand for 20 minutes, samples were centrifuged at 1000 G for 10 minutes at 25 °C, and the supernatant was kept at −80 °C until analysis. The menstrual fluid was collected using a menstrual collector (Menstrual Lunette Collector, Kevosai, Curitiba, Brazil), with each volunteer receiving a menstrual collector for individual use. Sampling was performed on the morning of the second day of menstruation, and the samples were collected at the analysis lab. The samples were immediately centrifuged for 10 minutes (1000 G), at 25 °C, and the supernatant was stored at −80 °C until analysis.

### Prostaglandins, enzymes involved inthe arachidonic acid pathway and pro-inflammatory cytokines in peripheral blood and menstrual fluid

PGF2α and PGE2 quantification in menstrual fluid, peripheral serum and supernatant were performed using KHL1731 and KHL1701 ELISA kits (Biosource, Caramillo, CA, USA) from samples diluted at 1:100 and 1:10, respectively. The pro-inflammatory cytokines: IL-1β, IL-6, TNF-α; and the enzymes: AKR1B1 and AKR1C3 were measured using CHC1213, CHC1263 and CHC1753 kits (Invitrogen-Caramillo, CA, USA) and LS-F10794 and LS-F14889 kits (LifeSpanBioSciences, Inc. Seattle, WA, USA) from samples diluted at a proportion of 1:10, always following the manufacturer’s instructions. The absorbance of the samples was detected using a monochromator (Asys UVM 340, Holliston, MA, USA) at a wavelength of 450 nm.

### Statistical analysis

Data were represented by the mean and standard error of the mean (SEM). In cases where the data followed a normal distribution, according to the Shapiro-Wilk’s test, differences between groups were determined using the Anova and Newman-Keuls statistic tests for multiple comparisons. When the normality could not be guaranteed, the data were analyzed by the Kruskal-Wallis non-parametric test accompanied by the Dunn´s test for multiple comparisons. For cases with two factors, Anova Two Way or Mann-Whitney U corrected by the Bonferroni’s method were employed. The software used for analysis was the GraphPad Prism version 5.00 for Windows (GraphPad Software, San Diego California USA). The tests were considered statistically significant when the *p-value* was less than 0.05.

### Ethical approval

All procedures performed in this study involving human participants were in accordance with the ethical standards of the institutional and/or national research committee (Comitê de Ética em Pesquisa em Seres Humanos da Universidade Federal de São Carlos http://www.propq.ufscar.br/etica/descricao-cep-n ° 48233715.8.0000.5504) and with the 1964 Helsinki declaration and its later amendments or comparable ethical standards.
